# The person behind the label: co-production as a tool in teaching about borderline personality disorder

**DOI:** 10.1192/bjo.2021.355

**Published:** 2021-06-18

**Authors:** Nyakomi Adwok, Sharon Nightingale

**Affiliations:** Leeds and York Partnership Foundation Trust

## Abstract

**Aims:**

The overarching aim of the session was to address and reduce stigma around Borderline Personality Disorder among doctors. The three main objectives were:

To increase empathy and understanding around Borderline Personality Disorder by exposing junior doctors to service user perspectives outside a clinical setting;

To address knowledge gaps identified by junior doctors in a self-reported questionnaire disseminated prior to the teaching session;

To offer junior doctors a basic psychological framework to base their assessment and formulation of service users with personality disorders.

**Background:**

‘Borderline Personality Disorder: The Person Behind the Label’ was the title of the first co-produced teaching session in the Leeds and York Partnership Foundation Trust (LYPFT). Prior to the teaching session, an online questionnaire was sent out to trainees. The results highlighted three key issues:

Negative attitudes towards service users with personality disorders;

Poor subjective knowledge of the psychological models of personality disorders;

Perception among trainees that they do not receive adequate training to deal with the challenges service users with personality disorders present.

**Method:**

A teaching session was co-produced by a team of two service users, a principal clinical psychologist within the Leeds Personality Disorder Network (PDN) and a core Psychiatry trainee. It was delivered in a 75 minute session to 40 attendees consisting of both trainee doctors and consultants.

**Result:**

Feedback was collected immediately after the session through the use of anonymous feedback forms. The response to the training was overwhelmingly positive with all 28 respondents rating the session as 4/5 or 5/5 on a satisfaction scale ranging from 1 (poor) to excellent (5). Key themes from the feedback included appreciation for the service user perspective and teaching on psychological theory. The fourth question in the questionnaire: “How will this teaching impact your work?” produced the highest number of responses (25/28) and provided evidence that the above listed objectives of the session were met.

**Conclusion:**

Co-produced teaching has great potential to address negative attitudes around highly stigmatised conditions by bridging the gap that often exists between service users and mental health professionals.

